# Associations of cardiovascular disease morbidity and mortality in the populations watching major football tournaments: A systematic review and meta-analysis of observational studies

**DOI:** 10.1097/MD.0000000000019534

**Published:** 2020-03-20

**Authors:** Huajun Wang, Lunchang Liang, Ping Cai, Jianli Zhao, Lan Guo, Huan Ma

**Affiliations:** a The People's Hospital of Luoding, Affiliated Luoding Hospital of Guangdong Medical University, Luoding; b Guangdong Provincial Key Laboratory of Malignant Tumor Epigenetics and Gene Regulation, Breast Tumor Center, Sun Yat-sen Memorial Hospital, Sun Yat-sen University, Guangzhou; c Guangdong Provincial Key Laboratory of Coronary Heart Disease Prevention, Guangdong Cardiovascular Institute, Guangdong Provincial People's Hospital, Guangdong Academy of Medical Sciences, Guangzhou, Guangdong, China.

**Keywords:** cardiovascular disease, cardiovascular morbidity, cardiovascular mortality, football

## Abstract

**Background:**

Whether or not increased hospitalizations and/or deaths due to cardiovascular disease during major football tournaments (MFTs) remains controversial. We undertook a systematic review and meta-analysis of published studies to assess the relationships of cardiovascular events and MFTs.

**Methods:**

Observational studies reporting relationship of cardiovascular disease morbidity and mortality with MFTs during the days of games or within 2 weeks after game season were included. Relative risk ratios (RR) with 95% confidence interval (CI) were pooled and analyzed using a random/fixed-effects model.

**Results:**

Nineteen cross-sectional observational studies that examined the association between MFTs and non-fetal cardiovascular events and mortality were found to be eligible from 3419 references, for inclusion in the systematic review and meta-analysis. Of the 10 studies reported hospitalizations due to non-fetal acute cardiovascular events, the pooled RR was 1.17 (95% CI 1.01–1.36). Of the 10 studies reported cardiovascular mortality the pooled RR was 1.03 (95% CI 1.00–1.05). Of the studies examining the mortality, 6 studies reported the lost or win of the national team. Pooling of four studies where their national teams lost the MFTs produced a RR for the mortality of 1.19 (95% CI: 1.09–1.30), and 4 studies of the 6 whose national teams won produced a pooled RR for cardiovascular mortality of 0.88 (0.79–0.98).

**Conclusion:**

This systematic review and meta-analysis showed an increased risk of hospitalization due to non-fetal acute cardiovascular events and cardiovascular mortality with watching MFTs.

## Abstract 2

Supplemental Digital Content is available in the text

## 1 Introduction

Acute cardiovascular events including death can be triggered by emotional stress.^[1,2]^ When stressful situations occur massively (eg, earthquakes,^[3]^ terrorist attacks,^[4]^ or war^[5]^), the incidence of acute cardiovascular events rises among individuals involved.^[6]^

Strong emotions can be provoked by major football tournaments (MFTs),^[7]^ MFTs were including: World Cup (WC). European Cup (EC). Australian football league. World cup qualifications and the rugby World cup. It has been suggested that these emotions might precipitate adverse cardiovascular events among game spectators.^[8,9]^ Some studies on the other hand have however found no relationship between MFTs and adverse cardiovascular events.^[10,11]^ The possible explanations for these differences are differences in study designs, outcomes, study period, study populations, and type of sporting events.

Reliable estimates of risks associated with MFTs are of value for effective public health services. One previous systematic review of observational studies showed a weak additional risk of acute cardiovascular events existed when populations watched MFTs.^[10]^ The authors concluded that due to the low study numbers and the wide confidence intervals (CIs) observed, the reported observation was no more than a random chance. However, the results of individual studies continue to draw attention since.^[12]^ We therefore undertook a new systematic review and meta-analysis to identify the relative risk of non-fetal cardiovascular events and mortality among populations watching MFTs.

## 2 Material and methods

### 2.1 Research question

Our research question has 2 folds:

(1) Does the relative risk of cardiovascular mortality, and/or
(2) Does the relative risk of hospitalization due to non-fetal cardiovascular events differ when the people watch MFTs compared to that of ordinary times when there was no MFTs.

## 3 Methods

### 3.1 Patient and public involvement

This study was approved by the Guangdong Provincial People's Hospital ethics committee. The patients and the public were not involved in setting the research question or the outcome measures, and no patients were involved in developing plans for the design or implementation of the study. We are unable to disseminate the results to study participants because of the nature of a meta-analysis.

### 3.2 Search strategy and selection criteria

PubMed, Embase, and Cochrane Library databases were searched from the earliest index date-December 2000 until the December 20, 2018 with no language restrictions provided by the abstracts in English. Cross-sectional observational studies (both prospective and retrospective) that described the association between watching MFTs and the cardiovascular morbidity or mortality were eligible for inclusion in the systematic review and meta-analysis. The following MeSH terms were used for the search; that is, (See Supplemental data 1): soccer, football, rugby, World cup, European cup, cardiovascular disease, acute coronary syndrome, angina, arrhythmias, cardiac, coronary disease, myocardial infarction, heart arrest, death, sudden, heart failure. Two reviewers independently sifted the search results for relevant titles and/or abstracts. The reference lists of all relevant articles were manually screened to find other potentially eligible studies. When a title and/or abstract met the study eligibility criteria but it did not provide sufficient data for this meta-analysis, the full text of the study was retrieved and reviewed. Editorials, letters to the editor, review articles, case reports and animal experimental studies were excluded. For studies reported both single-match and pooled estimates, we included only the pooled estimates in the present review. In addition, if only gender-specific estimates were reported, we calculated a pooled estimate for men and women combined.

### 3.3 Data extraction and quality assessment

Two investigators (HM and DZH) independently reviewed the eligible studies and extracted the data using a standardized excel file. For each study, data were extracted by one investigator and checked by another investigator for accuracy; disagreements were resolved by consensus.

For each article, the extracted data included: first author, publication year, football game type, relative risk comparison, outcome, geographical location, population, number of cases who were hospitalized due to cardiovascular disease or died, unadjusted or adjusted risk estimates and their 95% CIs, adjusted confounding factors, quality assessment, and any other information relevant to the two research questions.

The quality of each included study was independently evaluated by two investigators (HM and DZH) using the Newcastle-Ottawa Scale (NOS).^[13]^ The quality score of the studies was calculated based on three components as follows: selection of study groups (0–4 points), comparability of study groups (0–2 points), and ascertainment of the interest outcome (0–3 points). The scores ranged from 0 to 9 points, with a higher score indicating better methodological quality. Any disagreements were resolved by consensus.

### 3.4 Data analysis

The unadjusted and multivariable-adjusted relative risk (relative risk ratio (RR), Odds ratio (OR) or Hazard ratio (HR)) were used based on the data provided by the original studies to estimate the association between watching MFTs and non-fetal cardiovascular events or cardiovascular mortality in the population. If the relative risk value was not given, we calculated the unadjusted RR using original data (number of population of the studied place and prevalence rate) reported in the study. If the population size was not given, it was obtained by searching the most recent national census posted online. As the incidence of non-fetal cardiovascular events and cardiovascular mortality is relatively low, the OR or HR were assumed to be accurately close to the estimates of the RR.

Forest plots were used to show RRs and 95% CIs for the included studies. Overall RRs were calculated using fixed/random-effects models (DerSimonian and Laird method), potential heterogeneity among studies was calculated using the I^2^ statistic, which is a quantitative measure of inconsistency across studies. When I^2^ < = 50, fixed-effects model was used, when I^2^ > 50, random-effects model was used. Sensitivity and subgroup analyses were used to further explore study heterogeneity. Because only a few covariates were individually significant, a multivariate meta-regression model was not developed.

Publication bias was assessed by visual inspection of a funnel plot and the Begg and Egger tests. Subgroup analyses were done to test individual association of selected covariates with the pooled estimates. Subgroup analyses were grouped based on the following:

(1) The results of the MFTs (with the national team won the MFTs vs lose the MFTs);
(2) The type/region of the MFTs (World Cup (W.C.)/ European Cup (E.C) vs Australian football league, World cup qualifications, or the rugby World cup).
(3) Type of study (prospective vs retrospective). As mortality studies are all retrospective studies, this grouping method is only applicable to the morbidity studies.

A 2-tailed *P* value below .05 was considered statistically significant. All statistical analyses (except the Begg and Egger tests) were performed using Review Manager 5.0 and Stata 10.0. Begg and Egger tests were performed using Stata 12.0.

## 4 Results

### 4.1 Search results

**Figure 1:**
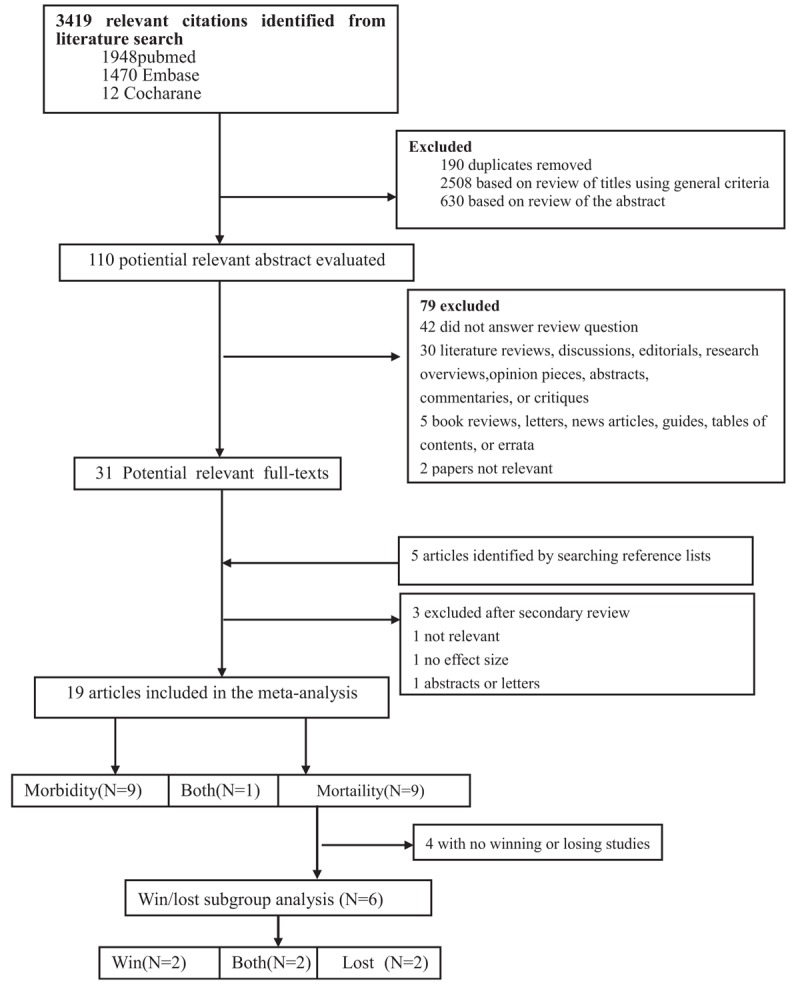
Flow chart of study selection.

Figure 1 shows our search strategy and results. Our initial literature search yielded 3419 abstracts (N = 2562) or titles (N = 857). Of these, 2508 were excluded as unsuitable after screening of the title; another 190 were excluded because of duplication. Furthermore, another 630 were excluded after the abstracts were screened. The full text was examined for the remaining 91 publications.

According to the predefined inclusion criteria, 20 publications met our criteria for this systematic review and meta-analysis.^[9–12,14–29]^ One of the chosen studies was presented in only abstract that was included^[25]^ because enough data provided in the abstract. Two of the 20 studies provided ORs, 14 provided RRs, and HRs provided in 2 studies. There were 2 studies did not provide relative risk estimates, therefore we calculated the RRs using the reported rates or raw data.^[14,28]^ For 1 study,^[29]^ we could not obtain neither OR, RR, or HR, because the data provided by the study could not yield for the relative risk estimate. Therefore, we did not include this study in this meta-analysis.

### 4.2 Study characteristics

Supplemental data 2 summarizes the features of the included publications (N = 19). Of them, nine publications presented cardiovascular mortality only,^[11,14–18,20,25,26]^ 9 presented hospitalizations due to non-fetal acute cardiovascular events only,^[9,10,12,19,21–24,28]^ 1 publication presented both conditions.^[27]^ Most studies (N = 14) were conducted in Europe; 3 were in Germany, 3 in Switzerland, 2 in England, 2 in France, 2 in Netherlands, 1 in Portugal and 1 in Italy. The rest were in USA (N = 2), Brazil (N = 1), New Zealand (N = 1), and Australia (N = 1). None were conducted in Africa or Asia. The populations involved in these studies ranged from 1.2 million to 193 million.

Of the studies examined the hospitalizations due to acute cardiovascular events, 4 were due to acute myocardial infarction,^[10,19,23,24]^ 1 was due to out-of-hospital cardiac arrests,^[21]^ 1 due to sudden cardiac death,^[22]^ and 4 studies included other forms of acute cardiovascular events.^[9,12,27,28]^ Of the 10 publications examined the cardiovascular mortality during the MFTs;^[11,14–18,20,25,26,29]^ 6 studies had the mortality due to acute myocardial infarction,^[11,15–18,20]^ 2 due to circulatory causes,^[14,26]^ 1 due to coronary heart disease,^[25]^ and 1 study examined the all-cause mortality.^[29]^

All the selected studies were considered in high quality with the median NOS score was 8 (ranging from 8 to 9).

### 4.3 Meta-analysis of cardiovascular risk

**Figure 2:**
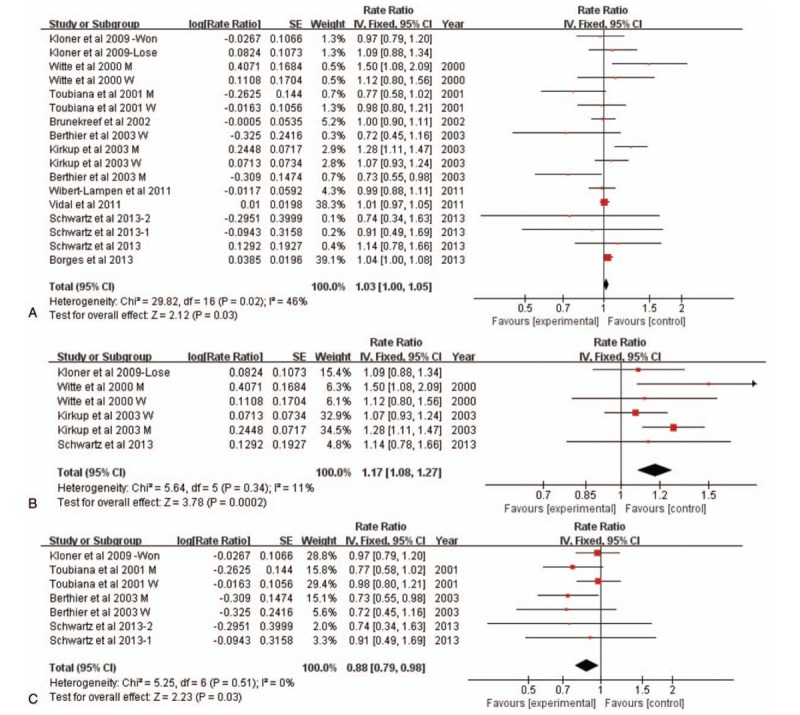
A. Forest plot of association between watching MFTs and the risk of cardiovascular mortality. B. Forest plot of association between losing MFTs by the populations’ team and the risk of cardiovascular mortality. C. Forest plot of association between winning football games by the population's team and the risk of cardiovascular mortality. MFTs = major football tournaments.

Analysis of the ten studies reporting cardiovascular mortality showed a significantly statistical association of watching MFTs with an increased risk of mortality, though the risk is small (RR 1.03; 95% CI: 1.00–1.05; Fig. 2A). The heterogeneity of the studies was moderate (I^2^ = 46%, *P* = .02). Analysis of subgroups showed that cardiovascular mortality was significantly increased among populations whose national team lost in the MFTs (RR 1.19; 95% CI: 1.09–1.30; Fig. 2B) and the risk of cardiovascular mortality was significantly lower in the populations whose national teams won (RR 0.88; 95% CI: 0.79–0.98; Fig. 2C). The heterogeneity of the lost team studies was minimum (I^2^ = 1%, *P* = .41). Similarly, the heterogeneity of the won team studies outcome was also minimum (I^2^ = 0%, *P* = .51). When the subgroup analyze was conducted based on the game type: W.C./ E.C (N = 7) vs non-W.C./E.C (N = 3), it showed that cardiovascular mortality was increased among non-W.C./E.C studies (RR 1.12; 95% CI: 1.03–1.21), with a minimum heterogeneity (I^2^ = 14%, *P* = .32). No significant association was found between watching W.C./ E.C games and the risk of cardiovascular mortality (RR 1.02; 95% CI: 0.99–1.04; I^2^ = 50%, *P* = .003)(Supplemental data 3).

**Figure 3:**
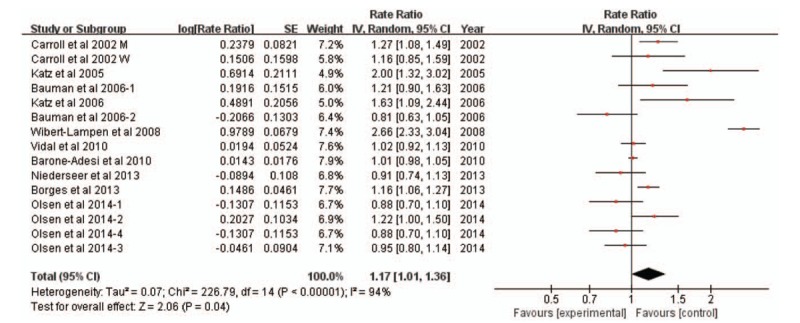
Forest plot of association between watching major football tournaments and the risk of hospital admission due to cardiovascular disease.

Our random-effects meta-analysis showed that watching MFTs was associated with a small and statistically increased risk of cardiovascular morbidity (RR 1.17; 95% CI: 1.01–1.36; Fig. 3). Significant heterogeneity was found among the studies (I^2^ = 94%, *P* < .00001). The subgroup analysis to better understand the cause of the high heterogeneity were shown in Supplemental data 3.

The sensitivity analysis revealed the combined RR of overall risk estimates were consistent and without apparent fluctuation. There was no publication bias found via the Begg and Egger tests (*P* > .05)

## 5 Discussion

This present systematic review and meta-analysis produced a couple of folds of findings regarding the relationship of the significant cardiovascular mortality and morbidity with the populations watching MFTs. Our results indicate that the incidence of cardiovascular mortality was affected by the final results of the MFTs, that is, the incidence became significantly great among the population whose teams lost the tournament (RR 1.19; 95% CI: 1.09–1.30), whereas the risk of cardiovascular mortality was significantly lower in the populations whose national teams won (RR 0.88; 95% CI: 0.79–0.98). These findings may be useful for public health planning organizations.

The only existing systematic review and meta-analysis by far that included 10 studies presented a range of estimated relative risk ratio of 0.7 and 1.3. The authors concluded that the cardiovascular effects of watching football matches are likely to be, if any, very small. Differently from that study, our presentation consisted of almost double size of the previous study, and separately analyzed the data based on mortality or morbidity. Furthermore, we specifically examined the impact of the final results of these MFTs on the cardiovascular outcomes. While the overall meta-analysis of our study support that watching MFTs have increased mortality, with the specific subgroup analysis, we found that home team winning the MFTs reduced the mortality at 12% and losing the MFTs increased the mortality of 19%. The findings suggest that the overall RR of the watching MFTs with mortality (RR = 1.03) was due to the combination of increased rate of mortality due to loss and the decreased mortality due to win.

**Figure 4:**
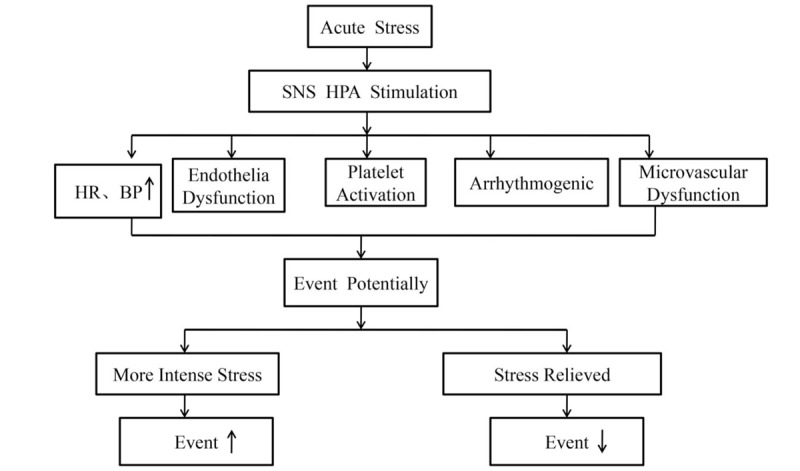
Proposed mechanisms of winning or losing the tournament affect cardiovascular mortality.

For passionate fans who identify with the home team, a dramatic and high-stakes game can be a stressful event, and winning or losing of the home team may bring different emotional reaction to the fans. Fans may become depressed and anger with the home team losses the tournament, whereas become euphoric and excited with the home team wins. Different type of emotional reaction may impact on the cardiovascular health differently. Depression and anger are prospectively associated with the development and progression of cardiovascular disease, which may worsen the already unstable cardiovascular system.^[30,31]^ There is consistent evidence that long-term depression and anger increase cardiovascular risk and may also acutely trigger cardiovascular events.^[32]^ Willich et al reported that emotional upset was associated with a transient 2.7-fold (95%CI: 1.1–6.6) higher risk of MI within 24 hours.^[33]^ And data from SHEEP study indicate that people were 9.0 (95% CI: 4.4–18.2) times more likely to experience an MI in the hour after episodes of anger than during other times.^[34]^ Positive emotions on the other side have been suggested to be associated with better cardiovascular outcomes. For instance, Laura et al found that positive psychological factors was associated with a decreased risk of combined angina, nonfatal MI, and CHD death.^[35]^ The meta-analysis by Christina et al reported that positive constructs were associated with reduced rates of hospitalization or mortality for cardiovascular disease patients.^[36]^ The findings of our present study support the speculation that when the home team win, the fans become happy and satisfied. Such positive emotional responses play a protective role on the cardiovascular system, which may promote restoration from “too tense state” and serve as “health assets”.^[37]^ Conversely, when the home team loss, the fans develop negative emotions that enhance “tense state” and reduce “health assets” (Fig. 4).

Our overall analysis showed a small and a significant increased risk of cardiovascular morbidity (RR1.17; 95%CI:1.01–1.36). When subgroup analyses conducted based on the research design, the results are far different. In the only prospective study, the incidence of hospitalizations due to acute cardiovascular events in a sample of German population during the 2006 World Cup season^[12]^ was close to 2.7-fold increase (RR 2.66; 95%CI:2.33–3.04) compared to the incidence during the non-season. However, the relative risk of the pooled analysis of the retrospective studies was across one (RR 1.07;95%CI:0.99–1.16). Compared with the retrospective study, prospective study is less susceptible to bias, and can calculate the incidence and accurately analyze the relative risk, which is generally considered more robust. Future studies examining the impact of watching MFTs on cardiovascular morbidity needs to be prospective in design.

## 6 Conclusions

This systematic review and meta-analysis showed an increased risk of hospitalization due to non-fetal acute cardiovascular events and cardiovascular mortality with watching MFTs. The cardiovascular mortality appeared to be related to the final results of the games, that is, winning the game had a positive impact and losing the game had a negative impact on mortality.

## 7 Limitation

The advantages of studying stressful triggers at a population-level are that the timing of exposure can be objectively identified and a population-based sampling frame prevents issues of selection bias that may arise in studies of triggers that are experienced by only a subgroup of the population. However, because aggregate population-level data were used in these MFTs studies, the observed associations may be due to individual-level confounding. Unlike wars and earthquakes, which can be assumed to affect the entire population, it may not be appropriate to assume that the people who experienced an acute cardiovascular event after a sports competition are the people who actually watched the event. Furthermore, these events may also involve multiple other stressors (lack of sleep, overeating, heavy alcohol ingestion, smoking, and failure to comply with medical regimes and so on), therefore it is difficult to isolate which, if any, are responsible for the empirically observed higher incidence. Distinguishing the different impact of these stressors can be very costly.

In addition, though nearly all studies including the process of adjustment for confounding factors when examining the relationships between watching METs and cardiovascular mortality and morbidity, the confounding variables such as the season, temperature, day of the week, or air pollution adjusted in each study were inconsistent. Comprehensively adjusting for various confounding factors is an important measure but is highly challenging.

In conclusion, our systematic review and meta-analysis showed that populations watching MFTs have increased risk of cardiovascular mortality when their home or favorable team lose the tournament. Conversely, winning a MFTs may be protective for the fans of the team from cardiovascular standpoint. Enhancing awareness of cardiovascular health in fans involving in MFTs may be necessary to prevent these individuals from getting into high negative emotional arousals.

## Acknowledgment

The authors thank Wei Jiang, MD, Departments of Internal Medicine and Psychiatry and Behavioral Sciences, Duke University Medical Center, Durham, North Carolina.

## Supplementary Material

Supplemental Digital Content

## Supplementary Material

Supplemental Digital Content

## Supplementary Material

Supplemental Digital Content

## References

[1] Esler M Mental stress and human cardiovascular disease. Neurosci Biobehav Rev 2016;192:37–137. 10.1016/j.neubiorev.2016.10.011 27751732

[2] KK GG Mental stress and cardiovascular disease: growing evidence into the complex interrelation between mind and heart. Angiology 2015;66:5–7. 24622932 10.1177/0003319714525032

[3] Takegami M Miyamoto Y Yasuda S Comparison of cardiovascular mortality in the great east japan and the great Hanshin-Awaji earthquakes- a large-scale data analysis of death certificates. Circ J 2015;79:1000–8. 25912562 10.1253/circj.CJ-15-0223

[4] Chi JS Poole WK Kandefer SC Cardiovascular mortality in New York city after September 11. Am J Cardiol 2003;92:857–61. 14516894 10.1016/s0002-9149(03)00901-9

[5] Bergovec M Mihatov S Prpić H Acute myocardial infarction infarction among civilians in Zagreb city area. Lancet 1992;339:303. 10.1016/0140-6736(92)91370-n 1346300

[6] Fontes MA Xavier CH Marins FR Emotional stress and sympathetic activity: contribution of dorsomedial hypothalamus to cardiac arrhythmias. Brain Res 2014;20:49–58. 10.1016/j.brainres.2014.01.043 24491632

[7] Schwartz BG French WJ Mayeda GS Emotional stressors trigger cardiovascular events. Int J Clin Pract 2012;66:631–9. 22698415 10.1111/j.1742-1241.2012.02920.x

[8] Murray A Mittleman EM Physical, psychological and chemical triggers of acute cardiovascular events preventive strategies. Circulation 2011;124. 10.1161/CIRCULATIONAHA.110.968776 PMC3139921 21768552

[9] Olsen PEJ Frampton C Bradley P Winning or losing does matter: acute cardiac admissions in New Zealand during rugby world cup tournaments. Eur J Prev Cardiol 2014;22:1254–60. 24924743 10.1177/2047487314539433

[10] Barone-Adesi F Vizzini L Merletti F It is just a game: lack of association between watching football matches and the risk of acute cardiovascular events. Int J Epidemiol 2010;39:1006–13. 20211850 10.1093/ije/dyq007

[11] Toubiana L French cardiovascular mortality did not increase during 1996 European football championship. BMJ 2001;322:1306. PMC1120392 11403063

[12] Wilbert-Lampen U Leistner D Greven S Cardiovascular events during world cup soccer. N Engl J Med 2008;358:475–83. 18234752 10.1056/NEJMoa0707427

[13] Stang A Critical evaluation of the Newcastle-Ottawa scale for the assessment of the quality of nonrandomized studies in meta-analyses. Eur J Epidemiol 2010;25:603–5. 20652370 10.1007/s10654-010-9491-z

[14] Kloner RA McDonald S Leeka J Comparison of total and cardiovascular death rates in the same city during a losing versus winning super bowl championship. Am J Cardiol 2009;103:1647–50. 19539070 10.1016/j.amjcard.2009.02.012

[15] Witte DR Bots ML Hoes AW Cardiovascular mortality in Dutch men during 1996 European football championship: longitudinal population study. BMJ 2000;321:1552–4. 11124170 10.1136/bmj.321.7276.1552 PMC27557

[16] Kirkup W Merrick DW A matter of life and death: population mortality and football results. J Epidemiol Community Health 2003;57:429–32. 12775788 10.1136/jech.57.6.429 PMC1732472

[17] Wilbert-Lampen U Nickel T Scheipl F Mortality due to myocardial infarction in the Bavarian population during world cup soccer 2006. Clin Res Cardiol 2011;100:731–6. 21431879 10.1007/s00392-011-0302-7

[18] Brunekreef B Hoek G No association between major football games and cardiovascular mortality. Epidemiology 2002;13:491–2. 10.1097/00001648-200207000-00022 12094109

[19] Carroll D Ebrahim S Tilling K Admissions for myocardial infarction and world cup football: Database survey. BMJ 2003;325:1439–42. 10.1136/bmj.325.7378.1439 PMC139028 12493655

[20] Berthier F Boulay F Lower myocardial infarction mortality in French men the day France won the 1998 world cup of football. Heart 2003;89:555–6. 12695467 10.1136/heart.89.5.555 PMC1767655

[21] Katz E Metzger JT Schlaepfer J Increase of out-of-hospital cardiac arrests in the male population of the French speaking provinces of Switzerland during the 1998 FIFA world cup. Heart 2005;91:1096–7. 16020610 10.1136/hrt.2004.045195 PMC1769050

[22] Katz E Metzger JT Marazzi A Increase of sudden cardiac deaths in Switzerland during the 2002 FIFA world cup. Int J Cardiol 2006;107:132–3. 16337515 10.1016/j.ijcard.2005.01.029

[23] Bauman AE van der Ploeg HP Chey T The hazards of watching football-are Australians at risk? MJA 2006;185:684–6. 17193725 10.5694/j.1326-5377.2006.tb00765.x

[24] Marques-Vidal P Paccaud F Watching football matches and the risk of acute myocardial infarction. Int J Epidemiol 2011;40:838–9. 21075810 10.1093/ije/dyq221

[25] Marques-Vidal P Nothing lethal: no relationship between euro/world football cup matches and increased CHD deaths in Switzerland. EuroPRevent Congress Abstracts 2011;p516:S108.

[26] Schwartz BG McDonald SA Kloner RA Super bowl outcome's association with cardiovascular deaths. Clin Res Cardiol 2013;102:807–11. 23979499 10.1007/s00392-013-0593-y

[27] Borges DG Monteiro RA Schmidt A World soccer cup as a trigger of cardiovascular events. Arq Bras Cardiol 2013;100:546–52. 23657272 10.5935/abc.20130105

[28] Niederseer D Thaler CW Egger A Watching soccer is not associated with an increase in cardiac events. Int J Cardiol 2013;170:189–94. 24182671 10.1016/j.ijcard.2013.10.066

[29] Medenwald D Kuss O Mortality on match days of the German national soccer team: a time series analysis from 1995 to 2009. J Epidemiol Community Health 2014;68:869–73. 24811776 10.1136/jech-2013-202844

[30] Jiang WB James A Depression and ischemic heart disease: overview of the evidence and treatment implications. Curr Psychiatry Rep 2003;5:47–54. 12686002 10.1007/s11920-003-0009-1

[31] Redford B Williams D Anger and mental stress-induced myocardial ischemia: mechanisms and clinical implications. Am Heart J 2014;169:4–5. 25497241 10.1016/j.ahj.2014.09.003

[32] Steptoe A Strike PC Perkins-Porras L Acute depressed mood as a trigger of acute coronary syndromes. Biol Psychiatry 2006;60:837–42. 16780810 10.1016/j.biopsych.2006.03.041

[33] Willich SN Lewis M Löwel H Physical exertion as a trigger of acute myocardial infarction: triggers and mechanisms of myocardial infarction study group. N Engl J Med 1993;329:1684–90. 8232457 10.1056/NEJM199312023292302

[34] Moller JHJ Hallqvist J Diderichsen F Do episodes of anger trigger myocardial infarction? A case-crossover analysis in the Stockholm heart epidemiology program (sheep). Psychosom Med 1999;61:842–9. 10593637 10.1097/00006842-199911000-00019

[35] Pulkki-Råback LEM Elovainio M Hakulinen C Positive psychosocial factors in childhood predicting lower risk for adult type 2 diabetes: the cardiovascular risk in Young Finns Study, 1980–2012. Am J Prev Med 2017;52:e157–64. 28284747 10.1016/j.amepre.2017.01.042

[36] DuBois CM Lopez OV Beale EE Relationships between positive psychological constructs and health outcomes in patients with cardiovascular disease: a systematic review. Int J Cardiol 2015;195:265–80. 26048390 10.1016/j.ijcard.2015.05.121 PMC4487518

[37] Boehm JK Kubzansky LD The heart's content: the association between positive psychological well-being and cardiovascular health. Psychol Bull 2012;138:655–91. 22506752 10.1037/a0027448

