# Supplementary material for: Associations of cardiovascular disease morbidity and mortality in the populations watching major football tournaments: A systematic review and meta-analysis of observational studies
Source: Medicine (Baltimore). 2020 Mar 20;99(12):e19534. doi: 10.1097/MD.0000000000019534 (PMC7220255; doi:10.1097/MD.0000000000019534)
Supplement: Supplemental Digital Content [file medi-99-e19534-s001.pdf]

Search strategy: Pubmed

ID	Search
#1	MeSH descriptor: [football] explode all trees
#2	MeSH descriptor: [soccer] explode all trees
#3	MeSH descriptor: [Cardiovascular Diseases] explode all trees
#4	MeSH descriptor: [Acute Coronary Syndrome] explode all trees
#5	MeSH descriptor: [Angina] explode all trees
#6	MeSH descriptor: [Arrhythmias] explode all trees
#7	MeSH descriptor: [Cardiac] explode all trees
#8	MeSH descriptor: [Coronary Disease] explode all trees
#9	MeSH descriptor: [Myocardial Infarction] explode all trees
#10	MeSH descriptor: [Heart Arrest] explode all trees
#11	MeSH descriptor: [Death] explode all trees
#12	MeSH descriptor: [Sudden] explode all trees
#13	MeSH descriptor: [heart failure] explode all trees
#14	rugby:ti,ab
#15	"World cup":ti,ab
#16	"European cup":ti,ab
#17	(#1 or #2 or #14 or #15 or #16)
#18	(#3 or #4 or #5 or #6 or #7 #8 or #9 or #10 or #11 or #12 #13)
#19	#17 and #18

Search strategy: Embase

ID	Search
#1	'football' /exp or 'football'
#2	'soccer' /exp or 'soccer'
#3	rugby:ab,ti
#4	'world cup'' :ab,ti
#5	'european cup' :ab,ti
#6	#1 or #2 or #3 or #4 or #5
#7	'Cardiovascular Diseases' /exp or 'Cardiovascular Diseases'
#8	'Acute Coronary Syndrome' /exp or 'Acute Coronary Syndrome'
#9	'angina pectoris' /exp or 'angina pectoris'
#10	'heart arrhythmia' /exp or 'heart arrhythmia'
#11	'coronary artery disease' /exp or 'coronary artery disease'
#12	'ischemic heart disease' /exp or 'ischemic heart disease'
#13	'heart infarction' /exp or 'heart infarction'
#14	'acute heart infarction' /exp or 'acute heart infarction'
#15	'heart failure' /exp or 'heart failure'
#16	'heart arrest' /exp or 'heart arrest'
#17	'sudden death' /exp or 'sudden death'
#18	#7 or #8 or #9 or #10 or #11 or #12 or #13 or #14 or #15 or #16 or #17
#19	#6 and #18

Search strategy: Coebrane

ID	Search
#1	MeSH descriptor: [football] explode all trees
#2	MeSH descriptor: [Soccer] explode all trees
#3	"Rugby":ti,ab,kw (Word variations have been searched)
#4	World cup:ti,ab,kw (Word variations have been searched)
#5	European cup:ti,ab,kw (Word variations have been searched)
#6	#1 or #2 or #3 or #4 or #5
#7	MeSH descriptor: [Cardiovascular Diseases] explode all trees
#8	MeSH descriptor: [Acute Coronary Syndrome] explode all trees
#9	MeSH descriptor: [Angina Pectoris] explode all trees
#10	MeSH descriptor: [Arrhythmias, Cardiac] explode all trees
#11	MeSH descriptor: [Coronary Disease] explode all trees
#12	MeSH descriptor: [Myocardial Infarction] explode all trees
#13	MeSH descriptor: [Heart Arrest] explode all trees
#14	MeSH descriptor: [Death] explode all trees
#15	MeSH descriptor: [Death, Sudden, Cardiac] explode all trees
#16	MeSH descriptor: [Heart Failure] explode all trees
#17	(#7or #8 or #9 or #10 or #11or # 12 or # 13 or # 14 or # 15 or #16)
#18	#6 and #17
