# Supplementary material for: Associations of cardiovascular disease morbidity and mortality in the populations watching major football tournaments: A systematic review and meta-analysis of observational studies
Source: Medicine (Baltimore). 2020 Mar 20;99(12):e19534. doi: 10.1097/MD.0000000000019534 (PMC7220255; doi:10.1097/MD.0000000000019534)
Supplement: Supplemental Digital Content [file medi-99-e19534-s002.pdf]

Study	Competition	Comparison	Outcome	Population (year:) million	N	RR(95% CI)	Won/ Lose	Adjustments
Witte[14] *et al.* 2000	E.C.（1996）	Events during the day of the last match played by the national team vs events during preceding and following 5 days	Mortality for AMI in The Netherlands	7.1	M:41<br>W:38	M: 1.51 (1.08–2.09)<br>W: 1.11 (0.8–1.56)	Lose	1,2,4
Toubiana[8] *et al.* 2001	E.C.（1996）	Events during the day of the last match played by the national team vs events during preceding and following 5 days	Mortality for AMI in France	58.0	M:61<br>W:83	M: 0.74 (0.58–1.02)<br>W: 0.96 (0.80–1.21)	Won	2,4
Brunekreef and Hoek[17] 2002	E.C.(1988 and 1992)<br>W.C.(1990 and 1994)	Events during the days of five matches played by the national team in the four tournaments vs events during other days between 1986 and 1994	Mortality for AMI in The Netherlands	6.7	NA	M+W: 1.00(0.90–1.11)	NA	1,2,3, 5,6,7
Carroll[9] *et al.* 2003	W.C. (1998)	Events within 2 days after a match of the national team vs events during the other days of the tournament	Hospital admissions for AMI in England	58.0	M+W:270	M:1.2(1.08-1.49)<br>W:1.16(0.85-1.59）	Lost	1,2,3,4,5
Berthier and Boulay[28] 2003	W.C. (1998)	Events during the day of the final match of the national team vs events during the preceding and following 5days	Mortality for AMI in France	58.3	M: 23<br>W: 18	M:0.71(0.55–0.98)<br>W:0.65(0.45–1.16)	Won	1,2,3,4
Kirkup and Merrick[15] 2003	E.F.T. (1994–1999)	Events on days when the local team lost at home vs events on days with any other result	Mortality for AMI in some UK health authorities	2.0	M: 220<br>W: 202	M:1.28(1.11–1.47)<br>W:1.07(0.93–1.24)	Lost	2,3,4
Katz[19] *et al.* 2005	W.C. (1998)	Events during days of the tournament vs events during the preceding 33days and following 33 days	Incidence of out-of-hospital cardiac arrests in selected provinces of Switzerland	1.5	M+W:45<br>M: 37<br>W: 8	M+W: 2.00(1.32–3.02)<br>M:2.31(1.40–3.83)<br>W:1.23(0.44–3.20)	NA	2,4
Katz[20] *et al.* 2006	W.C. (2002)	Events during days of the tournament vs events during the same period in 2001	Incidence of sudden cardiac death in selected provinces of Switzerland	1.5	M+W:38<br>M: 26<br>W: 12	M+W: 1.63(1.09–2.44)<br>M:1.77(1.09–2.86)<br>W:1.33(0.63–2.82)	NA	1,4
Bauman[21] *et al.* 2006	A.F.L (2005)<br>W.C.Q. (2005)	Events within 2 days after the AFL final match and after the regional World Cup	Hospital admissions for AMI in New South Wales (Australia)	6.8	(A.F.L) M+W:79<br>(W.C.Q) M+W:71	(A.F.L.) M+W: 1.21 (0.90–1.63)<br>(W.C.Q.) M+W: 0.81 (0.63–1.05)	NA	2,3

		qualifying match vs events during the preceding 14 days and following 11 days						
Wibert-Lampen[11] *et al*.2008	W.C. (2006)	Events during days of matches played by the national team vs events during control days in the years 2003,2005 and 2006	Hospital admissions for acute cardiovascular events in Bavaria (Germany)	1.8	M: 216<br>W: 86<br>M+W:302	M:3.26(2.78–3.84)<br>W:1.82(1.44–2.31)<br>M+W: 2.66(2.33–3.04)	NA	1,3,4,5,6,8
Kloner[13] *Et al*.2009	Super bowl （1980 and 1984）	Events during days and the following 14 days of matches played by the local team VS events during control days in the years 1980 to 1983 and 1984 to 1988	Mortality for all-cause and circulatory ，IHD，AMI，HF in LA （USA）	1980: 7.5	NA	1.09（0.88-1.34）	Lose	1,2,3
				1984 : 8.2	NA	0.97(0.79-1.20)	Won	
Barone-Adesi[7] 2010 Meta	E.C. (2004)<br>W.C.(2002)<br>W.C.(2006)	Events during the day of matches played by the national team vs events during the other days of the tournaments	Hospital admissions for AMI in Italy	2004: 57.4<br>2002: 57.4<br>2006: 58.8	M+W: 4395	M+W: 1.01(0.98–1.05)	NA	3
Vidal and Paccaud[22] 2011	E.C.(1996、2000、2004）<br>W.C.(2002)	Events during days of matches played by the national team vs events during the other days of the tournaments	Hospital admissions for AMI in Portugal	10.0-10.5	M+W:506	M+W: 1.02（0.92-1.13）	NA	1,3
Wilbert-Lampen[16] *et al*. 2011	W.C. (2006)	Events during days of matches played by the national team vs events during control days in the years 2003,2005 and 2006	Mortality for MI in Bavaria (Germany)	1.8	M：158<br>W：127<br>M+W:285	M:0.99 (0.84-1.15)<br>W:0.99 (0.82-1.19)<br>M+W: 0.99(0.88-1.11)	NA	1,2,3,4,5,8
P Marques-Vidal[25] 2011	E.C.(1980 to 2004)<br>W.C.(1982 to 2006)	Events during days of matches vs events during control days in the years 1980-2006	Mortality for CHD in Switzerland	6.3-7.6	NA	M:1.03(0.97 -1.09)<br>W:0.98(0.92-1.04)<br>M+W: 1.01 (0.96 - 1.05)	NA	1,4
Schwartz[26] *et al.* 2013	Super Bowls (2005-2009)	Eents during days of eight consecutive days beginning Super Bowl day VS events during the non-case period Super Bowls from 2005 to 2009.	Mortality for circulatory disease in Massachusetts, Arizona and Pittsburgh (USA)	Massachusetts : 6.5	NA	M+W: 1.14（0.78-1.66）	Lose	1,2,
				Arizona :3.75	NA	M+W: 0.91（0.49-1.69）	Won	
				Pittsburgh: 1.2	NA	M+W: 0.75（0.34-1.63）	Won	

Borges [27] *et al.*<br>2013	W.C.<br>(1998,2002,<br>2006,2010)	Events during days of matches played by the national team vs events during control days in the years 1998-2010	Hospital admissions for ACS and mortality in Brazil	166.3-193.3	AMI admission	M:1.16 (1.03-1.31)<br>W:1.16 (1.02-1.17)<br>M+W：<br>1.16 (1.06;1.27)	NA	1,4
					Mortality	M:1.14 (0.92;1.41)<br>W:1.09 (0.86;1.38)<br>M+W:<br>1.04(0.93-1.08)		
Niederseer [28]<br>*et al*.2013	W.C. (2006)	Events during days of matches played by the national team vs events during control days in the years 2005 and 2006	Hospital admissions for acute cardiovascular events in Bavaria (Germany)	12.6	NA	M+W:<br>0.91（0.74-1.13）	NA	1,2,4,5,6
Olsen [9]<br>*et al*.2014	RWC(1999)<br>RWC(2003)<br>RWC(2007)<br>RWC(2011)	Events during the days of national team's last match in each RWC and the 2 days after each match VSData for equivalent days for 3 years preceding 2011, 2007 and 2003 RWC or following 1999 RWC	Hospital admissions for ACSin New Zealand	1999: 3.8	NA	SWC 1999<br>M+W:<br>0.9（0.7-1.1）	Lose	1,2,3,4
				2003: 4.0	NA	SWC 2003<br>M+W:<br>1.2（1.0-1.5）	Lose	
				2007: 4.2	NA	SWC 2007<br>M+W:<br>0.9（0.8-1.14）	Lose	
				2011: 4.4	NA	SWC 2011<br>M+W:<br>0.9(0.7-1.1)	Won	

RRs refer to the numbers of cardiovascular events on days in which major football matches were played compared with events during control periods.

N: number of events during the exposure period; E.C.: European Championship; W.C: World Cup; E.F.T: English Football Tournament; A.F.L.: Australian Football League; W.C.Q: World Cup Qualifications; R.W.C: Rugby World Cup. M: men; W: women; M+W: both genders;Win: matches won by the team; Loss: match lost by the team; NA: not available.

Adjustments ：1: year; 2 :month; 3:day of the week; 4: gender; 5: temperature; 6: air pollution; 7: influenza epidemics.; 8: barometric pressure.
