# Supplementary material for: Associations of cardiovascular disease morbidity and mortality in the populations watching major football tournaments: A systematic review and meta-analysis of observational studies
Source: Medicine (Baltimore). 2020 Mar 20;99(12):e19534. doi: 10.1097/MD.0000000000019534 (PMC7220255; doi:10.1097/MD.0000000000019534)
Supplement: Supplemental Digital Content [file medi-99-e19534-s003.pdf]

Group	Subgroup	Categorical data	included studies (n)	Heterogeneity test (I^2, *P*)	Pooled RR(95% CI)
Mortality	The result of MFT	Win	4	11%，0.34	1.17（1.08-1.27）
		Lost	4	0%，0.51	0.88（0.79-0.98）
	Competition type	W.C./E.C.	7	92%, 0.0002	1.02(0.99-1.04)
		Non- W.C./E.C.	3	14%, 0.32	1.12(1.03-1.21)
Morbidity	The result of MFT	Win	3	0%，0.57	1.00（0.89-1.11）
		Lost	3	61%，0.04	1.10（0.94-1.28）
	Competition type	W.C./E.C.	6	96%, ＜0.00001	1.32 (1.07-1.63)
		Non- W.C./E.C.	2	53%, 0.06	0.98(0.86-1.12)
	Study design	Retrospective	7	60%, 0.004	1.07(0.99-1.16)
		Prospective	1	NA	2.66(2.33-3.04)
